# INSIGHT: An integrated framework for safe and sustainable chemical and material assessment

**DOI:** 10.1016/j.csbj.2025.03.042

**Published:** 2025-03-29

**Authors:** Angela Serra, Dimitrios Zouraris, Alexandra Schaffert, Marcella Torres Maia, Periklis Tsiros, Ishita Virmani, Emanuele Di Lieto, Laura Aliisa Saarimäki, Jack Morikka, Rafael Riudavets-Puig, Dimitra-Danai Varsou, Konstantinos D. Papavasileiou, Panagiotis D. Kolokathis, Dimitris G. Mintis, Haralampos Tzoupis, Andreas Tsoumanis, Georgia Melagraki, Alex Arvanitidis, Philip Doganis, Vasileios Minadakis, Giannis Savvas, Adrien Perello-y-bestard, Stefano Cucurachi, Marija Buljan, Fotini Nikiforou, Achilleas Karakoltzidis, Spyros Karakitsios, Dimosthenis A. Sarigiannis, Steffi Friedrichs, Christian Seitz, Tomas Navarrete Gutierrez, Panagiotis Isigonis, Sébastien Cambier, Antonino Marvuglia, Gottlieb Georg Lindner, Jacques-Aurélien Sergent, L. Cristiana Gheorghe, Laura-Jayne A. Bradford, Seung-Geun Park, Seung Min Ha, Zayakhuu Gerelkhuu, Tae Hyun Yoon, Romana Petry, Diego Stéfani Teodoro Martinez, David A. Winkler, Peter Wick, Thomas E. Exner, Francesco Dondero, Tommaso Serchi, Willie Peijnenburg, Haralambos Sarimveis, Martin Paparella, Iseult Lynch, Antreas Afantitis, Dario Greco

**Affiliations:** a Finnish Hub for Development and Validation of Integrated Approaches (FHAIVE), Faculty of Medicine and Health Technology, Tampere University, Tampere 33100, Finland; b Division of Pharmaceutical Biosciences, Faculty of Pharmacy, University of Helsinki, Helsinki 00790, Finland; c NovaMechanics Ltd, Nicosia 1070, Cyprus; d Entelos Institute, Larnaca 6059, Cyprus; e School of Chemical Engineering, National Technical University of Athens, Attiki 15772, Greece; f Medical University Innsbruck, Institute for Medical Biochemistry, Innsbruck 6020, Austria; g EMPA Materials Science and Technology, Lerchenfeldstrasse 5, St. Gallen CH-9014, Switzerland; h NovaMechanics MIKE, Piraeus 18545, Greece; i Division of Physical Sciences and Applications, Hellenic Military Academy, Vari 16672, Greece; j Institute of Environmental Sciences, Leiden University, P.O. Box 9518, Leiden 2300 RA, Netherlands; k Aristotle University of Thessaloniki, Department of Chemical Engineering, Environmental Engineering Laboratory, University Campus, Thessaloniki 54124, Greece; l HERACLES Research Center on the Exposome and Health, Center for Interdisciplinary Research and Innovation, Balkan Center, Bldg. B, 10th km Thessaloniki – Thermi Road, 57001, Greece; m AcumenIST SRL, Rue Fétis 19, Etterbeek 1040, Belgium; n Luxembourg Institute of Science and Technology (LIST), Luxembourg; o Evonik Operations GmbH, Research, Development & Innovation, Bruehler Strasse 2, Wesseling 50389, Germany; p Solvay SA, Toxicological and Environmental Risk Assessment Unit, Rue de Ransbeek 310, Bruxelles 1120, Belgium; q School of Geography, Earth and Environmental Sciences, University of Birmingham, Birmingham B15 2TT, United Kingdom; r Centre for Environmental Research and Justice, University of Birmingham, Edgbaston, Birmingham B15 2TT, United Kingdom; s Department of Chemistry, College of Natural Sciences, Hanyang University, Seoul 04763, South Korea; t Institute of Next Generation Material Design, Hanyang University, Seoul 04763, South Korea; u Brazilian Nanotechnology National Laboratory (LNNano), Brazilian Center for Research in Energy and Materials (CNPEM), Campinas, Sao Paulo, Brazil; v Department of Biochemistry and Chemistry, La Trobe Institute for Molecular Science, La Trobe University, Bundoora, Victoria 3086, Australia; w Monash Institute of Pharmaceutical Sciences, Monash University, Parkville, Victoria 3052, Australia; x School of Pharmacy, University of Nottingham, Nottingham NG7 2RD, United Kingdom; y Seven Past Nine d.o.o., Hribljane 10, Cerknica 1380, Slovenia; z Department of Science and Technological Innovation, University of Eastern Piedmont, Alessandria 15121, Italy; aa National Institute for Public Health and the Environment (RIVM), Center for Safety Assessment of Substances and Products, Bilthoven, Netherlands

**Keywords:** Safe and sustainable by design (SSbD), Impact outcome pathway (IOP), Non-animal or new approach methods (NAMs), FAIR principles, Chemical and material risk assessment

## Abstract

The assessment of chemicals and materials has traditionally been fragmented, with health, environmental, social, and economic impacts evaluated independently. This disjointed approach limits the ability to capture trade-offs and synergies necessary for comprehensive decision-making under the Safe and Sustainable by Design (SSbD) framework. The EU INSIGHT project addresses this challenge by developing a novel computational framework for integrated impact assessment, based on the Impact Outcome Pathway (IOP) approach. Extending the Adverse Outcome Pathway (AOP) concept, IOPs establish mechanistic links between chemical and material properties and their environmental, health, and socio-economic consequences. The project integrates multi-source datasets (including omics, life cycle inventories, and exposure models) into a structured knowledge graph (KG), ensuring FAIR (Findable, Accessible, Interoperable, Reusable) data principles are met. INSIGHT is being developed and validated through four case studies targeting per- and polyfluoroalkyl substances (PFAS), graphene oxide (GO), bio-based synthetic amorphous silica (SAS), and antimicrobial coatings. These studies demonstrate how multi-model simulations, decision-support tools, and artificial intelligence-driven knowledge extraction can enhance the predictability and interpretability of chemical and material impacts. Additionally, INSIGHT incorporates interactive, web-based decision maps to provide stakeholders with accessible, regulatory-compliant risk and sustainability assessments. By bridging mechanistic toxicology, exposure modeling, life cycle assessment, and socio-economic analysis, INSIGHT advances a scalable, transparent, and data-driven approach to SSbD. This project aligns with the European Green Deal and global sustainability goals, promoting safer, more sustainable innovation in chemicals and materials through an integrated, mechanistic, and computationally advanced framework.

## Graphical Abstract

**Figure:**
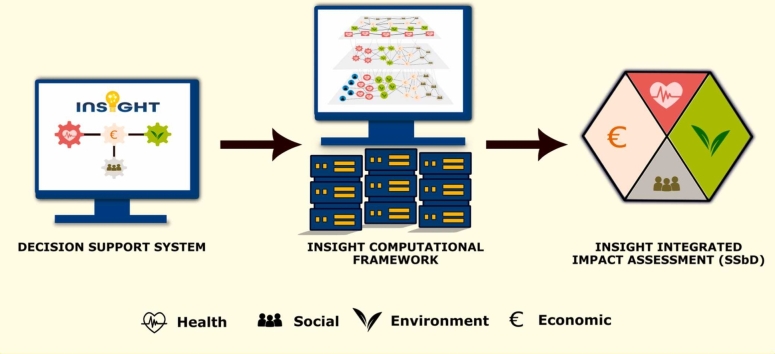

## 1 Introduction

The safety and sustainability of chemicals and materials have been at the forefront of global environmental and health concerns over the past decade. Ever-increasing numbers of new chemicals are released into the environment without a deep understanding of their broader health, (eco)toxicological, environmental, and socioeconomic impacts [1], [2]. Addressing this gap is a cornerstone of the EU Green Deal (https://commission.europa.eu/strategy-and-policy/priorities-2019–2024/european-green-deal_en), which aims for zero pollution of air, water, and soil by 2050. The Chemicals Strategy for Sustainability (CSS) serves as a key instrument in this initiative, aiming to phase out harmful substances and promote safer alternatives. However, current challenges remain, including unsustainable production and consumption patterns, as well as a lack of data for comprehensive safety and sustainability assessment. A truly holistic assessment must integrate eco- and human toxicity, climate impact, biodiversity, and socio-economic factors.

The reliance on animal testing in human and ecotoxicological regulation conflicts with the Green Deal goals of sustainability for society, economy and ecosystems [3]. Furthermore, current methods often yield unreliable results for humans and ecosystems, while outdated testing approaches and ethical concerns hinder the timely assessment of thousands of existing and new, ‘greener’ chemicals [3]. New Approach Methodologies (NAMs) offer a path forward to advance sustainability and foster economic opportunities like green chemical innovation, by enabling faster, more ethical, and resource-efficient chemical assessment. However, their successful regulatory application requires improved validation processes and data integration tools [4].

The Safe and Sustainable by Design (SSbD) framework aims to bridge these gaps by embedding safety and sustainability considerations of human health and the environment throughout the entire lifecycle of chemicals and materials [5]. The SSbD framework adopts an iterative approach, incorporating safety, sustainability, and circular economy considerations from the earliest stages of chemical and materials development. Central to the SSbD framework are the four key dimensions: health, environment, social, and economic impacts. While these dimensions have long been known to be critical for chemical safety, they are often assessed independently using different methodologies and data sources. Safety is mainly addressed by the risk assessment framework, environmental sustainability by Life Cycle Assessment (LCA), social impacts are characterised by Social Life Cycle Assessment (S-LCA) and economic impacts by Life Cycle Costing (LCC). Such fragmentation hinders fully integrated and holistic decision-making, the lack of which is becoming increasingly critical as industries strive to comply with stricter regulatory standards [6] and address growing public demand for chemicals that are safe and sustainable for people and the environment. To be fully effective, SSbD must move beyond this current fragmentation and enable a unified assessment framework that integrates safety and sustainability metrics, ensuring consistent and data-driven decision-making throughout the chemical and material life cycle. The INSIGHT project aims to provide a solution by developing a novel platform for chemical impact assessment. This platform will allow an integrated impact assessment and will use the concept of the Impact Outcome Pathways (IOP) to elucidate the effects of chemicals and materials across safety, social, economic and environmental domains.

## 2 Project description

### 2.1 Project scope and objectives

**Fig. 1:**
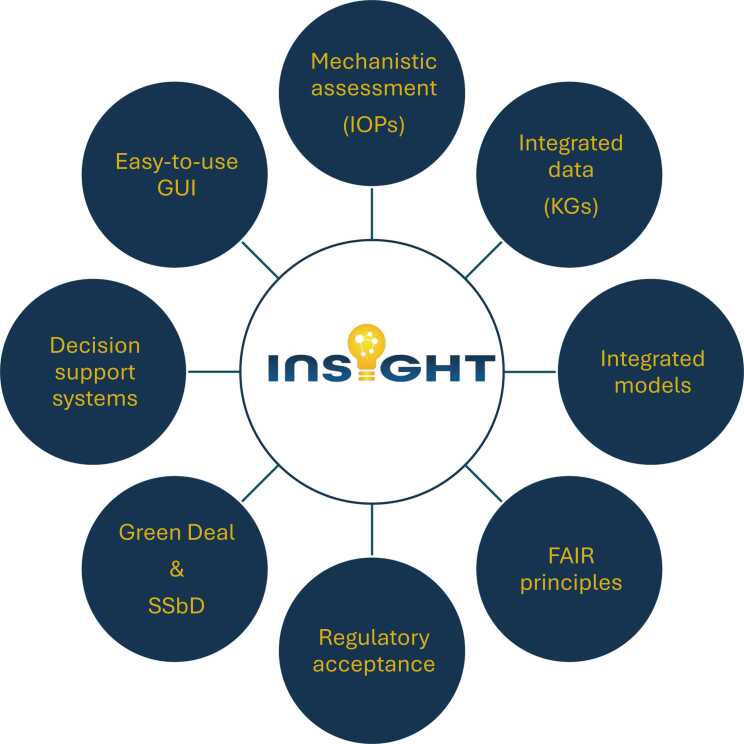
Overview of the INSIGHT project aims. FAIR: Findable, Accessible, Interoperable, Re-usable; GUI: Graphical User Interface (for all tools and models) to enable non-programming experts to utilise the models easily and effectively. IOPs: Impact Outcome Pathways. KGs: Knowledge Graphs.

The INSIGHT project aims to develop an integrated, mechanistic framework for assessing chemical and material impacts across environmental, health, social, and economic dimensions (Fig. 1).

#### 2.1.1 A novel mechanistic impact assessment framework

Central to the INSIGHT project’s mechanistic assessment framework is the novel concept of Impact Outcome Pathway (IOP), which extends the Adverse Outcome Pathway (AOP) concept [7] beyond its current scope to encompass social, economic and environmental aspects. AOPs are being increasingly applied in toxicology to establish causal links between molecular perturbations and adverse biological effects, significantly enhancing regulatory science by providing mechanistic insight into chemical hazards [8], [9]. However, AOPs remain limited to human and environmental health and do not extend to broader sustainability considerations, such as life cycle impacts, economic viability, and societal trade-offs. While life cycle assessment (LCA) and multi-criteria decision analysis (MCDA) have been employed for sustainability evaluations, these methods typically lack mechanistic explanations of the impacts. The INSIGHT project overcomes these limitations by introducing the IOPs, an extension of AOPs that systematically incorporates cross-domain interactions and enables mechanistic, multi-criteria decision-making. By establishing structured, causally linked pathways, IOPs will provide a framework that captures how chemical, and material properties drive interrelated environmental, economic, and social outcomes, facilitating holistic impact assessments.

#### 2.1.2 Multi-layered computational framework for integrated assessments

**Fig. 2:**
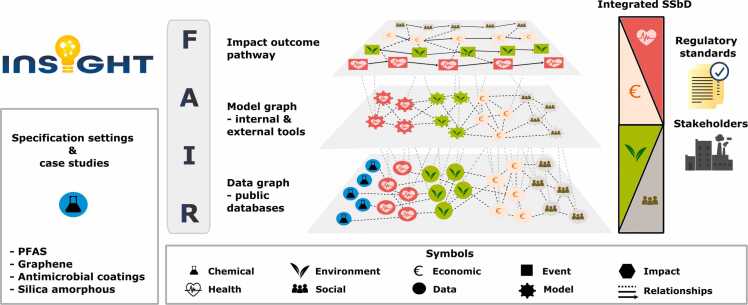
Schematic illustration of INSIGHT’s R&I strategy: the project will support stakeholders such as policymakers and industry actors in their decision-making process. At its core, the framework includes extensive collections of high-quality data that will be used by multiple models to integratively assess the health, ecological, social, and economic impacts of a chemical or material.

Essential to the INSIGHT approach is a multi-layered system that integrates data, computational models, and IOPs within a structured graph (Fig. 2), aligning with recent advancements in knowledge representation such as Knowledge graphs that have been increasingly used in the biomedical field [10], [11]. The data layer of INSIGHT’s multi-layered system serves as a centralized repository linking diverse datasets, including chemical properties, toxicological profiles, omics data, exposure metrics, and sustainability indicators. The harmonized curation and processing of this data ensures standardized, cross-domain integration. The model layer then processes this data using standardized procedures for omic data analysis, quantitative structure-activity relationship (QSAR) models, physiologically based kinetic (PBK) models, LCA, and socio-economic impact models, establishing predictive linkages between material characteristics and real-world effects. While previous efforts have developed predictive toxicology models and machine-learning-based sustainability assessments, these tools often operate in isolation, limiting their ability to provide a mechanistic understanding of multi-domain interactions. INSIGHT’s IOP foundation will synthesise these outputs into structured, causally linked pathways, mapping cascading effects across environmental, health, and economic systems, thereby improving interpretability and regulatory applicability. Ensuring FAIR (Findable, Accessible, Interoperable, and Reusable) [12] compliance, INSIGHT promotes transparent and reproducible assessments that support industry adoption and regulatory decision-making, aligning with recent initiatives in computational toxicology and cheminformatics that emphasize open data and model transparency.

#### 2.1.3 Data-driven decision support for regulatory and industrial application

This framework will be further operationalized with interactive decision-support tools that facilitate user-friendly, scientifically validated assessments, enabling industry stakeholders, policymakers, and regulatory bodies to more easily navigate complex datasets and modeling outputs when operating the framework. These tools will provide structured workflows that facilitate the systematic assessment of safety and sustainability based on multi-dimensional mechanistic criteria. Unlike conventional methodologies, which often require specialized expertise in multiple domains, INSIGHT’s tools will offer a user-oriented interface, allowing stakeholders to navigate complex data and models. The framework will be tested and refined through case studies (Fig. 2) focusing on per- and polyfluoroalkyl substances (PFAS), antimicrobial coatings, and graphene-based materials, ensuring applicability across different sectors and providing evidence-based insights into chemical and material impact trade-offs. By integrating structured mechanistic knowledge across multiple domains, the INSIGHT project will establish a transformative paradigm for chemical and material impact assessment, positioning IOPs as a novel approach that bridges the mechanistic insights of AOPs with the comprehensive scope of LCA and MCDA, providing an adaptive, scientifically rigorous framework for sustainability-focused decision-making. This contrasts starkly with traditional, fragmented assessments, creating a new and much-needed paradigm for comprehensive, lifecycle-based evaluations [13]. This proactive approach shifts the paradigm from isolated, reactive assessments to a comprehensive proactive strategy involving collaboration across academia, industry and regulators.

##### 2.1.3.1 Project consortium

**Fig. 3:**
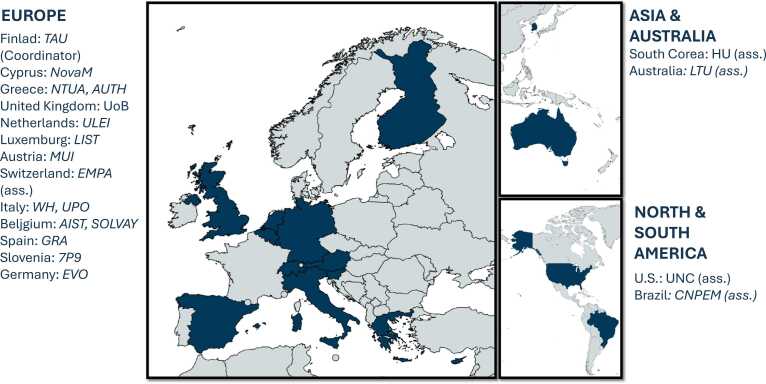
INSIGHT partners. Global distribution of project partners and associated collaborators within the INSIGHT project. The map highlights the participating institutions across Europe, Asia, Australia, and the Americas. In Europe, the partners include Tampere University (TAU, Coordinator) in Finland, NovaMechanics (NovaM) in Cyprus, the National Technical University of Athens (NTUA) and Aristotle University of Thessaloniki (AUTH) in Greece, the University of Birmingham (UoB) in the United Kingdom, Leiden University (ULEI) in the Netherlands, the Luxembourg Institute of Science and Technology (LIST) in Luxembourg, the Medical University of Innsbruck (MUI) in Austria, the Swiss Federal Laboratories for Materials Science and Technology (EMPA, Associate) in Switzerland, Warrant Hub (WH) and the University of Eastern Piedmont (UPO) in Italy, Acumenist (AIST) and Solvay (SOLVAY) in Belgium, Graphenea (GRA) in Spain, the 7P9 (7P9) in Slovenia, and Evonik Operations GmbH (EVO) in Germany. In Asia and Australia, the collaborators are Hanyang University (HU, Associate) in South Korea and La Trobe University (LTU, Associate) in Australia. In North and South America, the institutions include the University of North Carolina (UNC, Associate) in the United States and the Brazilian Center for Research in Energy and Materials (CNPEM, Associate) in Brazil.

The INSIGHT consortium comprises leading institutions and companies across multiple disciplines and regions with synergistic skills for creating an integrated, advanced framework to assess the safety and sustainability of chemicals and materials (Fig. 3). The project is coordinated by Tampere University (TAU) in Finland, which oversees the overarching scientific and technical integration of all research activities. The consortium’s partners National Technical University of Athens (NTUA), the University of Birmingham (UoB), Leiden University (ULEI), Aristotle University of Thessaloniki (AUTH), the University of Eastern Piedmont (UPO), and the Luxembourg Institute of Science and Technology (LIST) each offer complementary strengths in data science, LCA, and regulatory toxicology. The University of North Carolina at Chapel Hill (UNC) and La Trobe University (LTU) contribute to the development of computational models, supporting the mechanistic prediction of chemical hazards and exposure pathways. UoB and ULEI lead efforts in socio-economic, environmental, and health impact assessments. The Medical University of Innsbruck (MUI) and the Swiss Federal Laboratories for Materials Science and Technology (Empa) contribute knowledge in regulatory science and materials assessment as well as the bridge to a broad network of regulatory and policy stakeholders.

In addition to academic institutions, the consortium is strengthened by specialised Small and Medium Enterprises (SMEs) and research-intensive companies that drive technical development and data management. Novamechanics Limited and Seven Past Nine d.o.o. bring leading expertise in AI-driven data modelling and FAIR data principles that play a critical role in building INSIGHT’s computational models. Graphenea, Evonik Operations GmbH, and Solvay are industrial partners contributing to case studies on advanced 2D materials, antimicrobial materials, synthetic amorphous silica, and PFAS. International partners such as Hanyang University (HU) in South Korea and the Brazilian Center for Research in Energy and Materials (CNPEM) provide a global perspective that broadens the framework’s applicability across regulatory and industrial contexts worldwide. Warrant Hub and AcumenIST (AIST) support INSIGHT with expertise in regulatory frameworks, and technology transfer, ensuring that INSIGHT’s outputs align with policy requirements and meet industry standards.

##### 2.1.3.2 Project structure and work packages

**Fig. 4:**
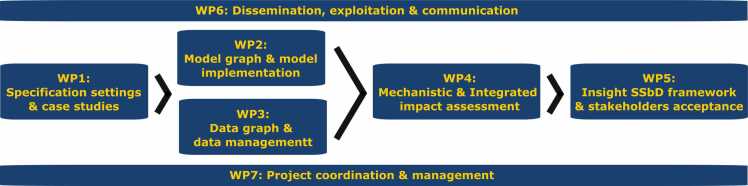
WP Structure of the INSIGHT Paper.

The INSIGHT project structure comprises seven interdependent work packages (WPs, Fig. 4), each contributing to a comprehensive SSbD assessment framework for chemicals and materials.

1. WP1 establishes the foundation by defining the specifications of four case studies: PFAS, 2D carbon nanomaterials (graphenes), amorphous silica (bio-based vs synthetic) and innovative nano-enabled antimicrobial substances. It defines requirements, data flows, and key performance indicators (KPIs), mapping socio-economic, health, and environmental impacts to ensure a precise focus for research. This groundwork supports the objectives of WP2, WP3, and WP4, ensuring each addresses data gaps and industry relevance.
2. WP2 develops a model graph, the computational core of INSIGHT, by implementing and benchmarking a comprehensive array of existing and new models. WP2 depends on the harmonised, FAIR-compliant data infrastructure established in WP1 and WP3.
3. WP3 curates, structures, and FAIRifies datasets, creating an accessible and interoperable resource for human and machine use. WP2 and WP3 form the essential modelling and data infrastructure, enabling WP4 to perform integrated impact assessments.
4. WP4 synthesises the outputs from WP2 and WP3 to create an advanced assessment framework, based on novel IOPs that consider social, economic, health, and environmental impacts in a mechanistic, integrated approach. This framework translates scientific models and data into a practical SSbD system that informs decision-making processes.
5. WP5 focuses on usability and stakeholder accessibility, developing an interactive graphical user interface (GUI) that enables industry and regulatory stakeholders to apply the SSbD framework in practice. WP5 also plays a critical role in refining the framework by incorporating stakeholder feedback, ensuring that INSIGHT’s outputs align with industry and regulatory needs.
6. WP6 oversees dissemination, exploitation, and communication, ensuring INSIGHT’s results are shared widely and understood across scientific, regulatory, and industrial sectors.
7. WP7 handles project management and coordination, facilitating cross-consortium communication and ensuring the project progresses smoothly and meets all milestones.

INSIGHT is funded through the European Union’s Horizon Europe programme under the call HORIZON-CL4–2023-RESILIENCE-01, which focuses on industrial resilience and sustainability. Managed by the European Health and Digital Executive Agency (HADEA), the funding comprises a grant of €4,130,318.75, supporting the project from January 2024 to December 2027. The Horizon Europe funding framework facilitates innovation and research aligned with EU priorities, such as the European Green Deal, and fosters collaborations aimed at sustainable and digital transitions across European industries. In addition to EU funding, contributions from partner countries further strengthen the project’s financial and research capacity. Switzerland and the United Kingdom provide direct support through their national funding mechanisms, while additional contributions from South Korea, Brazil, the United States, and Australia further enhance the project’s global reach. INSIGHT’s structure and funding model enable it to achieve meaningful advances in safe and sustainable chemical use, with the resources, expertise, and international reach needed to meet the project’s ambitious goals. Through coordinated efforts across diverse organisations, INSIGHT aims to make a lasting impact on how chemicals and materials are evaluated and managed, contributing to a safer, more sustainable future aligned with European and global priorities.

## 3 Case studies

The INSIGHT project aims to develop an integrated in silico framework that demonstrates the applicability of advanced computational modeling for chemical and material safety and sustainability assessment. This will be achieved through a series of case studies that have already been defined in WP1, each designed to address distinct classes of chemicals and materials, industrial applications, regulatory challenges, and methodological innovations. Specifically, the case studies are structured to (1) assess different classes of chemicals and materials, including Per- and Polyfluoroalkyl Substances (PFAS), Graphene-based materials (GBMs), Synthetic Amorphous Silica (SAS) and Antimicrobial coatings, (2) cover diverse industrial sectors, (3) integrate distinct computational models and tools to ensure seamless data flow and model interoperability, (4) evaluate regulatory compliance with frameworks such as Registration, Evaluation, Authorization, and Restriction of Chemicals (REACH) and the European Sustainability Reporting Standards (ESGR), and (5) explore scientific and regulatory questions that require the integration of previously disparate modeling approaches.

A unifying feature across all case studies is the informatics-driven approach, wherein diverse computational models, including exposure modeling, LCA, and toxicokinetic simulations, are integrated within a holistic assessment framework. A key innovation of the INSIGHT project is the use of a KG to structure and interlink disparate datasets, ensuring that chemical safety, environmental sustainability, and socio-economic impact assessments are seamlessly connected. By advancing computational modeling, regulatory science, and FAIR data principles, the INSIGHT case studies will demonstrate the transformative potential of *in silico* approaches in modern chemical and material risk assessment. The results will provide novel scientific insights and practical decision-support tools for regulators, industry stakeholders, and researchers, reinforcing the role of digital frameworks in the transition toward safe and sustainable materials.

### 3.1 Per- and polyfluoroalkyl substances (PFAS): integrating omics for comprehensive assessment

PFAS are widely utilized in lubricants and industrial applications due to their thermal stability, low friction properties, and resistance to degradation [14], [15]. However, their environmental persistence and bioaccumulative potential have raised significant regulatory concerns, particularly regarding contamination of water sources and potential health risks [16]. This case study will investigate computational approaches to identify safer alternatives to PFAS in lubricant formulations, integrating predictive toxicology models, environmental fate modeling, and exposure simulations. The regulatory framework governing PFAS use is evolving, with the European Union increasingly restricting their application through REACH and the Water Framework Directive. The INSIGHT framework will explore strategies to assess the feasibility of PFAS replacements while ensuring comparable functional performance. A mechanistic understanding of the interactions between PFAS and biological systems is crucial within the context of SSbD. To achieve this, PFAS omics data have already been systematically collected and curated during the initial phase of the project. To integrate these molecular insights into mechanistic assessments, structured frameworks are necessary to translate omics-derived signatures into meaningful toxicological predictors. In this context, partner TAU has developed and published an open dataset linking genes to key events (KEs) and adverse outcome pathways (AOPs) from the AOP-Wiki (https://aopwiki.org/) [17], [18]. This resource enables a mechanistic linkage between omics-derived molecular signatures and higher-level toxicological outcomes, enhancing the predictive capacity of computational models in regulatory toxicology. A fundamental analytical component of the case study is the systematic integration of models, for the analysis of the collected data, to evaluate PFAS properties, biological activity, and exposure pathways. An extensive literature review has identified relevant physics-based models, such as physiologically based kinetic (PBK) models, and machine learning models, including quantitative structure-activity relationship (QSAR) models, for predicting PFAS toxicity. These models will be integrated to build pipelines for integrated impact assessment. By combining external exposure information, in vivo biokinetics modelling and mechanistic insights from omics data, this framework provides a detailed mapping of dose-response relationships and predicts adverse outcomes of PFAS exposure in humans and ecosystems. These data and models will be systematically linked into the three-layer INSIGHT graph to perform mechanistic impact assessment through the IOP framework.

### 3.2 Graphene-based materials (GBMs) in concrete: a 2D materials perspective

GBMs have garnered significant interest in materials science due to their high surface area, sheet-like, and thin structure that contributes to outstanding electrical and thermal conductivity, optical properties, and mechanical strength [19]. These characteristics make GBMs a promising candidate for various applications in environmental remediation [20], energy storage and production [21], construction [22], drug-delivery and imaging [23]. In the construction sector, GBMs have been used as a reinforcing filler to enhance the functionality of cement, improving mechanical and thermal properties, and overall durability of concrete [22], [24]. However, key challenges remain, particularly regarding the scalability of high-quality GBMs production and the uncertainties surrounding their toxicity and environmental impact [19].

This case study aims to evaluate the impact of GBMs on composite material durability and recyclability, leveraging computational models to assess material degradation, environmental fate, and potential exposure scenarios. The regulatory landscape for GBMs includes chemical safety assessments under REACH, waste management policies, and environmental impact regulations within the EU Restriction of Hazardous Substances Directive (RoHS).

This case study focuses on the chemical safety of GBMs, in particular graphene oxide (GO), as a graphene reinforcement in concrete. With the data collected and the analyses conducted thus far, a preliminary analysis of the potential of GO in concrete was performed using a comprehensive pipeline of assessment tools. These included the Stoffenmanager Nano and Licara Nanoscan platforms, which provide insights into worker safety and the risk-benefit analysis of nanoproducts. Notably, no detectable airborne particles were observed during worker exposure assessments for GBMs. The social impacts of GO utilisation are being investigated through Social Life Cycle Assessment (S-LCA). Early findings suggest that most social risks are linked to background processes such as raw material procurement and waste management. Future analyses will refine the S-LCA approach and initiate an economic impact analysis. Environmental assessments are centred on the SimpleBox4Nano model to estimate predicted environmental concentrations (PEC) across multiple compartments, complemented by benchmark dose modelling (BMD) to derive risk characterisation ratios (RCR). Additionally, omics data integration will play a pivotal role, with RNA-seq and microarray datasets enabling the derivation of molecular-level BMDs and linking of overall gene expression alterations to AOPs. This will eventually allow the association of biological events described in the AOP to impact categories relevant for SSbD.

### 3.3 Synthetic amorphous silica (SAS): exploring omics data to understand exposure impacts

Synthetic amorphous silica (SAS) is commonly used as a reinforcing filler in tire manufacturing to improve performance and durability. The transition to bio-based SAS, derived from renewable agricultural waste, presents an opportunity to enhance sustainability by reducing the carbon footprint of tire production. However, ensuring consistent quality and scalable production remains a critical challenge. This case study will apply life-cycle assessment (LCA) and social-LCA methodologies to compare the environmental and economic impacts of bio-based SAS relative to conventional SAS. Additionally, the study will explore regulatory compliance pathways under the EU End-of-Life Vehicle (ELV) Directive and other environmental standards governing automotive materials. By integrating multi-model simulations, the INSIGHT framework will assess the feasibility of bio-based SAS as a scalable, sustainable alternative.

### 3.4 Antimicrobial coatings: UV-C up-converting materials and surfaces

Antimicrobial coatings are increasingly employed in healthcare, food packaging, and consumer products to prevent microbial contamination and enhance hygiene standards. However, their widespread application raises concerns regarding microbial resistance, potential toxicity, and environmental persistence. This case study will evaluate the comparative performance of nano-enabled antimicrobial coatings versus conventional disinfectants, focusing on leaching rates, degradation mechanisms, and long-term environmental impacts. Computational modeling will be used to simulate exposure pathways, assess toxicity profiles, and predict antimicrobial resistance potential. Regulatory oversight of antimicrobial coatings falls under the EU Biocidal Products Regulation (BPR), which mandates rigorous testing for safety, efficacy, and environmental sustainability. The integration of predictive modeling into regulatory decision-making will provide quantitative insights into the trade-offs between efficacy and long-term safety.

## 4 Integrated assessment framework

### 4.1 Model graph for integrated impact assessment of chemicals and materials

A major challenge in advancing SSbD is integrating the impact assessment of chemicals and materials across safety, environmental, and socio-economic dimensions. Traditionally assessed separately, these aspects require a holistic approach to capture trade-offs and synergies within a unified framework. To address this, WP2 aims to collect, develop, integrate, and FAIRify models critical for SSbD assessment. A key step for such advancement is the systematic collection of publicly available models aligned with SSbD requirements and indicators derived from the Joint Research Centre (JRC) reports on SSbD [5], [25]. This compilation includes a diverse range of models, including pipelines for omics data analysis [26], environmental fate, exposure, PBK, and QSAR, selected to address a broad range of dimensions of chemical safety and sustainability.

The INSIGHT partners have so far identified approximately 100 distinct models, each documented with its scope, applicability domain, and relevance to the case studies. These models are being integrated into a model graph, where models function as nodes and input-output compatibilities define the edges. Unlike traditional impact assessment modeling approaches, the model graph enables dynamic definition of the pipelines, based on data and model availability. Depending on the query, different subnetworks can be activated to generate relevant insights, ensuring that different impact dimensions are properly addressed in an integrated manner. This capability makes the model graph a key enabler of SSbD operationalization, as it allows chemical and material assessment to move beyond isolated evaluations toward a holistic decision-support system.

In some cases, the information flow within the model graph is bidirectional, for instance, PBK models can use internal concentrations to estimate external exposure and vice versa, depending on whether they are used in reverse or forward dosimetry mode [27]. The graph facilitates not only the sequential transformation of information through computational pipelines but also consensus modeling, where multiple models generate the same type of information, which is then processed by a single meta-node [28]. A critical aspect of modeling, particularly for regulatory purposes, is managing inherent prediction variability and uncertainty. Regulatory decision-making requires confidence in model predictions, yet individual models often have high uncertainty due to limited data or underlying assumptions. To address this, whenever possible, each model of the model graph will be accompanied by metrics that capture its associated uncertainty. Depending on data and model availability, techniques such as consensus-based modelling will be used to address model uncertainty. Modeling pipelines will also incorporate uncertainty propagation from input to output by defining well-characterized probability distributions for inputs and propagating stochasticity to the output at each node using sampling methods like Monte Carlo simulations [29]. This robust uncertainty quantification not only supports probabilistic risk assessment but also enhances decision-making by offering a clearer understanding of potential outcomes.

To support model interoperability and seamless integration, efforts are being conducted in designing and developing application programming interfaces (APIs) across the project’s framework. This setup allows models to operate with or without a graphical user interface (GUIs), supporting a range of user needs and technical capabilities. APIs are being designed for key models, including: BMDx [30] – a tool for benchmark dose modeling, crucial for dose-response analysis and hazard characterization; AOPFingerprint [17] – a tool that facilitates the linking of mechanistic toxicology data to AOPs, enhancing predictive toxicology [31], [32], [33]; INTEGRA [34]– a model enabling comprehensive environmental fate and exposure assessments, essential for risk assessment and regulatory compliance. To facilitate ease of access and deployment, models are hosted on platforms such as Jaqpot (https://jaqpot.org/), and integrated into the Enalos Cloud Platform (https://www.enaloscloud.novamechanics.com/insight.html), enabling flexible deployment through REST APIs and Docker containers. Jaqpot is an online platform for hosting machine learning and PBK models. The INSIGHT-dedicated Jaqpot instance provides free access to a collection of QSAR and PBK models. Software development kits (SDKs) for Python, Java, and R enable users to interact with Jaqpot's API, facilitating model integration into computational pipelines. This cloud-based infrastructure ensures that SSbD assessments remain scalable and accessible to a wide range of stakeholders, from regulatory agencies to industry practitioners.

**Fig. 5:**
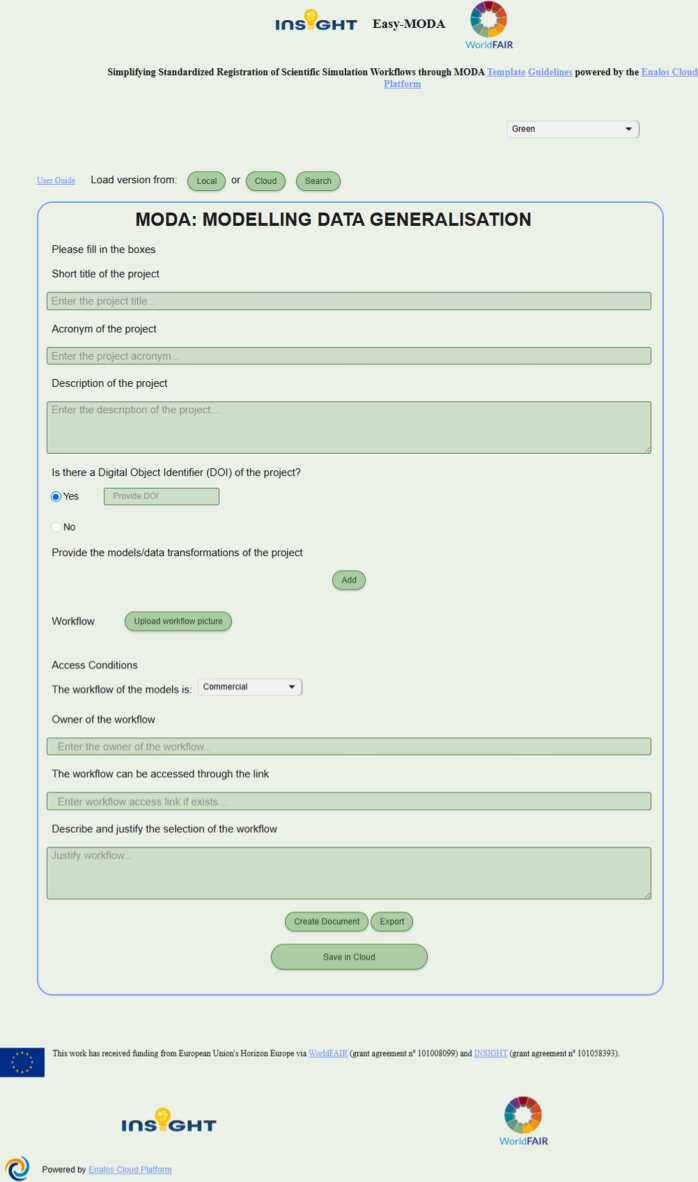
Easy-MODA hosted on the ENALOS Cloud Platform.

Consistent with FAIR principles, FAIR-ifying all models is essential in the INSIGHT framework. The team has implemented Easy-MODA, a tool that facilitates model documentation according to MODA guidelines [35]. This documentation standard supports transparency and reproducibility, providing detailed reporting for each model’s input, process, and output. Easy-MODA is hosted on the Enalos Cloud Platform [36], where users can generate MODA reports that systematically detail modelling workflows, thus streamlining both user engagement and data management across the platform (Fig. 5).

MODA documentation is only one part of the substantial effort made by INSIGHT to ensure that the models developed within the framework comply with the FAIR principles. To promote its SSbD characteristics and to contribute to the broader goal of harmonisation with the platform developed within INSIGHT, a systematic methodology was devised to evaluate and address any potential shortcomings in the models' compliance with the FAIR principles. To this end, a questionnaire was devised which includes several questions categorised based on the four FAIR pillars. Regarding Findability, these questions assess whether the models and associated metadata have been assigned globally unique and persistent identifiers (e.g., DOIs). Questions pertaining to Accessibility evaluate the retrievability of models and metadata using standardised communication protocols. With respect to Interoperability, this section of the questionnaire investigates the ability of the models to exchange information using established standards. Finally, questions regarding Reusability, determine whether the models offer tutorials, comprehensive documentation, and licensing terms. The questionnaire is used as a diagnostic tool to systematically find areas where individual models’ FAIR compliance is lacking. Each models’ compatibility with the INSIGHT platform can be enhanced by implementing targeted efforts to fill these gaps and improve alignment with the FAIR principles. By ensuring FAIR compliance, we further strengthen the foundation for an integrated impact assessment framework that is not only scientifically robust but also transparent and reproducible.

### 4.2 Data graph and data management processes

Effective impact assessment for chemicals and materials requires the integration of diverse and heterogeneous data sources, spanning experimental, computational, and bibliographic datasets [37]. However, current data management strategies are often fragmented, limiting the ability to capture the complex interdependencies between chemical properties, toxicological effects, and environmental impact. This challenge necessitates the development of an integrated data resource that ensures interoperability, contextualization, and seamless retrieval of relevant information. To address this, WP3 is building a structured KG tailored to impact assessments. KGs are graph databases where relevant entities are represented by nodes and associations or relationships between these entities are represented by their edges [10]. KGs also enable holistic representation of multi-dimensional chemical impact assessments by interconnecting structured datasets through relational nodes, making them a powerful tool for capturing non-linear relationships across diverse domains of chemical safety and sustainability assessments.

WP3 has made significant progress in establishing a robust data management and integration framework tailored to support SSbD assessments. A key milestone was the development and ongoing refinement of the Data Management Plan (DMP), a living document that guides consortium-wide data handling practices, ensuring adherence to FAIR principles and enabling seamless data interoperability. The structured nature of the DMP not only supports data organization but also facilitates its integration into predictive models, allowing for systematic uncertainty quantification and dynamic updates as new information becomes available. To further advance the FAIRification of data, the data graph under development links structured datasets via relational nodes, capturing interdependencies between chemical properties, toxicological profiles, and environmental impacts. This approach thus facilitates complex queries and efficient data retrieval.

Integrating multi-omics data into a structured KG is essential for advancing SSbD by addressing the fragmentation of chemical safety data. To demonstrate this, transcriptomics data from PFAS and GBM were curated and will be integrated into the INSIGHT KG, enabling mechanistic interpretation and predictive modeling. For PFAS, 20 datasets covering 26 compounds and 53 exposure studies were collected, primarily focusing on hepatotoxicity in human liver models, while environmental data remained limited with available studies primarily conducted in fish models. Similarly, 11 datasets for GBMs were retrieved, covering 16 materials across 23 exposure studies. Over half of these studies encompassed multiple doses allowing for dose-response modeling. Most of these studies support health hazard assessment using human-related models, such as *Homo sapiens*, *Rattus norvegicus*, and *Mus musculus*. In contrast, only a limited number of studies addressed environmental hazards, primarily relying on fish and nematode models representative of aquatic and soil systems. For SAS, 5 GEO datasets covering 9 distinct exposure studies were retrieved. The datasets encompass various tissues, species, and exposure types, offering a comprehensive view of SAS impacts. Human-based models included tissues such as blood, bone marrow, liver, and pancreas. Harmonized metadata, curated via ESPERANTO [15], ensures cross-study comparability and supports mechanistic insights by linking transcriptomic responses to AOPs. Differential expression analysis was performed using standard RNA-seq tools (e.g., HISAT2, *DESeq2* library in R) and microarray analysis tools (e.g., *limma* package in R, eUTOPIA shiny app). Previously developed gene-KE-AOP annotations will be leveraged to analyse PFAS related gene expression to identify possible Adverse Outcomes (AOs) associated with exposure. Furthermore, the BMDx tool will be used to identify points of departure (PoDs) for PFAS at the molecular level [16]. Transcriptomics data will be incorporated into the INSIGHT data graph along with PFAS relationships with genes, diseases, and pathways from the Comparative Toxicogenomic Database (CTD) that provides curated datasets. Also, apical endpoints described in other databases like ECOTOX and PubChem bioassays will be added.

Beyond structured databases, scientific literature provides an additional layer of knowledge critical for comprehensive chemical safety assessment. However, extracting relevant information from vast, unstructured text sources remains a significant challenge. In INSIGHT, novel exploratory approaches leveraging artificial intelligence (AI) and Large Language Models (LLMs) will be used to extract relevant associations between chemicals of interest and safety, environmental social and economic relevant impact and indicators. By applying AI-driven text mining, relevant exposure-outcome relationships from scientific literature (e.g., PubMed-indexed studies) can be extracted and categorized. This complements structured data sources by identifying emerging hazard evidence, including rare or underreported effects. Furthermore, a targeted approach will also be implemented to quantify co-occurrences of predefined terms associated with specific impact categories (e.g., human health, ecological risk, socio-economic sustainability). By combining structured database information with AI-extracted insights, this integrative strategy enhances the predictive power of the knowledge graph, enabling dynamic updates as new evidence emerges.

Finally, the database infrastructure, overseen by NovaMechanics, has been established through the Pharos Database Solution (https://pharos.novamechanics.com/), enabling flexible data upload, FAIR compliance, and integration into computational workflows. Pharos is also used to store additional (meta)data, e.g. full details on the experimental conditions, which are too extensive to be integrated into the KG. Planned activities include further expanding the KG, testing data representation techniques, and refining edge prediction algorithms to improve the predictive capabilities of the KG.

### 4.3 Integrating impact assessments aligned with SSbD principles

Current impact assessment methodologies mostly operate in isolation, lacking integration of mechanistic insights with environmental, social, and economic factors within the SSbD paradigm. To address this gap, INSIGHT WP4 is developing Impact Outcome Pathways (IOPs) as a novel extension of the existing Adverse Outcome Pathway (AOP) framework. IOPs expand beyond the toxicological endpoints of AOPs to include broader sustainability considerations, providing a structured, mechanistic foundation for assessing chemical and material impacts. Existing impact assessment methodologies often operate in silos and do not consider mechanistic interpretations. To bridge this gap, INSIGHT designs computational pipelines to integrate IOPs into structured, interoperable workflows that enable automated, mechanistic impact assessment.

These pipelines are designed to integrate life cycle inventories (LCIs) with broader life cycle impacts associated with the production, use, and disposal phases of chemicals. A recent workshop introduced partners to the USEtox characterisation model, highlighting its applications in assessing human health and ecotoxicity impacts within the LCA framework. This model is currently being adapted to conform to the Environmental Footprint (EF) 3.1 standards, providing a foundation for calculating LCA impact scores while encompassing additional environmental impact factors such as climate change, acidification, and other key impact categories.

WP4 has also commenced work on specific case studies to apply and enhance these models, focusing on priority chemicals like PFAS and nanomaterials (NMs). For instance, WP4 is developing improved *in silico* models for PFAS to predict fate and effect properties that will aid the assessment of aquatic toxicity across multiple species. These models employ machine learning to produce species sensitivity distributions (SSDs) for PFAS and predict physicochemical behaviours. Additionally, WP4 is developing AI-driven models to evaluate the aquatic toxicity of metal-based NMs across diverse ecological endpoints, leveraging data integration for enhanced predictive accuracy.

A major challenge in computational impact assessment is ensuring that mechanistic models and decision-support tools are not only scientifically rigorous but also accessible to regulatory bodies, industry stakeholders, and policymakers. To address this, WP4 will develop decision-support systems that translate high-dimensional computational outputs into structured, user-friendly guidance. The final implementation of these decision-support systems will be integrated into the INSIGHT portal developed in WP5, ensuring seamless usability through a web-based interface.

### 4.4 Aligning the SSbD framework with regulatory standards

**Fig. 6:**
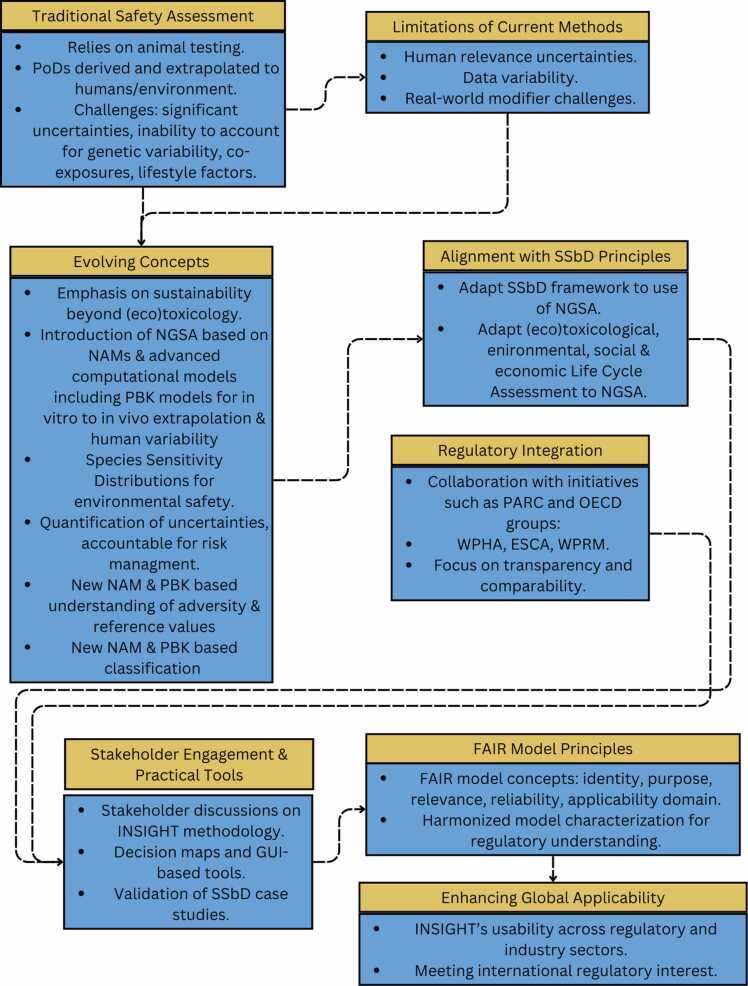
Aligning the SSbD framework with regulatory standards schematic representation.

WP5, the final technical INSIGHT WP, focuses on aligning the SSbD framework with regulatory standards, ensuring stakeholder acceptance, and developing accessible tools for practical implementation. This requires building on current regulatory concepts, identifying their limitations and uncertainties, and evolving the concepts towards sustainability (Fig. 6): Traditional safety assessments rely on animal testing to derive PoDs that are then extrapolated to humans or the environment using pragmatic, deterministic factors. This approach has significant uncertainties regarding relevance to humans, data variability, and the inability to account for real-world modifiers like genetic variability, co-exposures, or lifestyle factors. Non-animal methods (NAMs), combined with advanced computational models such as PBK models, allow target extrapolation and provide a quantification of uncertainties across PoD derivation, extrapolation, and target variability. In environmental safety assessment, NAMs including computational approaches, such as Species Sensitivity Distributions (SSD), may offer a more refined alternative to the simplistic use of single-animal-species derived PoDs and fixed assessment factors for acute and chronic toxicity. The integration of these approaches into a Next-Generation Safety Assessment (NGSA) framework supports the transition from outdated animal-based approaches to mechanistic, data-driven assessments aligned with SSbD principles. However, successful implementation requires stakeholder agreement, probabilistic modeling, and harmonised toxicological data for transparent and comparable decision-making.

To facilitate regulatory integration, INSIGHT has established collaborative links with key initiatives such as the partnership for the assessment of risks from chemicals (PARC - https://www.eu-parc.eu/), and other related research consortia. Engagement with expert groups of the organisation for Economic Cooperation and Development (OECD) such as the Working Party for Hazard Assessment (WPHA), the Advisory group on Emerging Science in Chemicals Assessments (ESCA), and the Working Party for Risk Management (WPRM) were initiated for future collaboration and discussion of the INSIGHT methodology. These interactions will be focused on the case studies, the envisaged decision maps, and the practical GUI based tools.

Furthermore, WP5 has progressed in the documentation and validation of SSbD case studies. Commonalities of the OECD-method characterisation formats for QSARs, NAMs, and PBK models were identified with a view to clustering the specific information requirements into five common concepts of validity, i.e. FAIR model identity, purpose, relevance, reliability and applicability domain (Fig. 6). This structured approach may increase readability and understanding for regulators while informing the development of new formats for omics data interpretation as needed [35].

## 5 Discussion

The early developments of the EU INSIGHT project indicate its potential to address longstanding challenges in chemical and material safety by integrating mechanistic, socio-economic, and environmental impacts. Although still in its initial phase, the introduction of IOPs as an extension of the AOP concept offers a novel means to link diverse impact dimensions that are often assessed in isolation. This fragmentation limits the ability to comprehensively evaluate trade-offs between safety, environmental, and socio-economic factors, which may result in decision-making that lacks a mechanistic basis and fails to capture the full life cycle implications of chemicals and materials.

One of the most significant INSIGHT outcomes is the establishment of a computational framework that follows FAIR principles to ensure the accessibility and interoperability of data. Tools such as the Jaqpot (https://jaqpot.org/) platform, the NovaMechanics Enalos Cloud Platform (https://www.enaloscloud.novamechanics.com/insight.html), combined with APIs for models like AOP fingerprinting [17] and BMDx [30], allow for integration of omics data and predictive modelling. These tools could provide a robust foundation for real-time, data-driven evaluations, which are often lacking in conventional assessment frameworks.

The early work on case studies demonstrates the potential of the INSIGHT framework to offer a more integrated and mechanistic approach to impact assessment. For example, in the GO case study, combining worker exposure data from Stoffenmanager Nano (https://nano.stoffenmanager.com/) with environmental fate models yielded comprehensive insights into occupational and ecological risks, thereby addressing nano-specific concerns not covered in standard OECD guidelines. Similarly, the PFAS case study highlights the importance of multi-omics integration in toxicological assessments. The systematic collection and curation of omics data from 53 exposure studies provide a valuable starting point for identifying molecular-level toxicity mechanisms and linking them to observed adverse outcomes. This contrasts with previous assessments that often relied on incomplete or aggregated datasets, limiting the granularity of insights. The SAS case study further illustrates the potential of curated omics data in enhancing exposure impact assessment, but similar challenges exist regarding data standardization and extrapolation to broader exposure scenarios.

Compared to existing methodologies, such as traditional LCA, INSIGHT promises integrated impact assessment with mechanistic contextualization. This could refine current impact assessment methodologies by improving the causal understanding of toxicity and sustainability trade-offs.

Nonetheless, difficulties lie in ensuring regulatory acceptance and scalability of emerging computational tools. For example, while the integration of omics datasets provides detailed mechanistic insights, the validation and standardisation of these datasets for regulatory purposes requires further refinement. Additionally, the emphasis on a limited set of case studies, such as GO, PFAS, and SAS presents the need for expanded applications across other chemical classes to ensure the generalisability of the INSIGHT framework. Moving forward, further efforts to align computational outputs with regulatory frameworks and extend the applicability of the tools to a broader range of chemicals will be essential. By doing so, INSIGHT can play a pivotal role in advancing the European Green Deal’s goals of safety, sustainability, and innovation in the chemical sector.

### 5.1 Dissemination and communication of the project’s results

The INSIGHT project’s dissemination and communication strategy ensures the effective sharing of its findings and the engagement of a wide range of stakeholders. The project website (www.insight-project.org) and Linkedin page (https://www.linkedin.com/company/insight-ssbd/) are the key dissemination routes to maximise its impact. In the first year of the project, 4 scientific publications in peer-reviewed, open-access journals have been published. These include:

• "A Network Toxicology Approach for Mechanistic Modelling of Nanomaterial Hazard and Adverse Outcomes" [32] introduces a novel framework to determine the mechanism of action of NMs,
• “Nanomaterial grouping: unraveling the relationship of induced mechanisms and potency at a temporal scale" which proposes a novel grouping strategy to allow robust hazard assessment of NMs [31],
• “The FAIR Principles as a Key Enabler to Operationalize Safe and Sustainable by Design Approaches" [38] highlighting the critical role of the FAIR principles in promoting safe and sustainable methodologies, and
• "Easy-MODA: Simplifying Standardised Registration of Scientific Simulation Workflows Through MODA Template Guidelines Powered by the Enalos Cloud Platform" [35] presents streamlined approaches to standardising the description (metadata) for simulation workflows to support broader accessibility.

Dissemination of project outcomes and outputs at relevant conferences has also been a key focus, with partners actively participating in international meetings to present project advances. These include the BioNanoNet Meeting in March 2024, where preliminary findings were shared, and the EUSAAT Conference in September 2024, which highlighted the role of novel methodological frameworks such as NGRA and SSbD in reducing and replacing animal testing. Additionally, events such as the PARERE Interactions in May 2024 and Materials Week in June 2024 enabled further outreach to regional authorities and research communities. The use of social media campaigns and website updates has enhanced communication. For instance, the International Day of Girls and Women in Science in February 2024 and regular project updates on Graphenea’s blog have ensured global accessibility to the project’s milestones and objectives. Stakeholder engagement has also been prioritised through targeted workshops and meetings, such as the FAIRification workshop in January 2025 and collaborations with the OECD to align project outcomes with regulatory frameworks. Looking ahead, INSIGHT plans to participate in upcoming conferences such as Eurotox 2025 and host additional workshops to disseminate its findings further and engage with the global scientific community, thereby consolidating its impact on advancing safe and sustainable design principles.

### 5.2 Future directions

In the next phase of the project, INSIGHT will focus on refining its computational and data integration capabilities and prioritising the FAIRification of datasets and models. Upcoming efforts include developing robust APIs for tools like INTEGRA and the LCA calculation framework Brightway (https://docs.brightway.dev/en/latest/), expanding the model graph, and integrating omics and PBK models to enhance predictive power for case studies such as PFAS and graphene. Addressing challenges like *in vitro*-to-*in vivo* extrapolation and long-term exposure modelling will remain central. Stakeholder usability will be improved through a user-friendly GUI that incorporates decision maps to guide SSbD assessments. SaaS deployment and stakeholder workshops will ensure regulatory and practical relevance, while collaboration with OECD expert groups will align the framework with international standards. The construction of a knowledge graph tailored to case studies will allow advanced data queries and predictive analytics, addressing critical data gaps.

## 6 Conclusion

As the demand for safer and more sustainable chemicals and materials continues to grow, the need for integrated, mechanistically informed assessment frameworks becomes increasingly urgent. The INSIGHT project advances this transition by integrating mechanistic modeling, life cycle impact assessment, and predictive computational tools into a unified framework. This approach moves beyond traditional, fragmented methodologies by systematically linking molecular mechanisms to environmental and human health outcomes. INSIGHT enhances both the transparency and applicability of SSbD principles through the development of interoperable data infrastructures and the incorporation of regulatory needs. Embedding the computational framework into user-friendly decision-support tools and expanding case study applications will further lay the groundwork for regulatory frameworks that not only assess safety but also drive innovation toward sustainability. Ultimately, this project represents a critical step toward replacing outdated evaluation models with dynamic, data-driven approaches that enable safer, more sustainable chemical and material design.

## CRediT authorship contribution statement

**Savvas Giannis:** Writing – review & editing, Writing – original draft. **Perello-y-bestard Adrien:** Writing – review & editing, Writing – original draft, Methodology. **Cucurachi Stefano:** Writing – review & editing, Funding acquisition, Methodology, Writing – original draft. **Buljan Marija:** Writing – review & editing, Writing – original draft. **Melagraki Georgia:** Writing – review & editing, Writing – original draft. **Arvanitidis Alex:** Writing – review & editing, Writing – original draft. **Doganis Philip:** Writing – review & editing, Writing – original draft. **Minadakis Vasileios:** Writing – review & editing, Writing – original draft. **Tzoupis Haralampos:** Writing – review & editing, Writing – original draft. **Tsoumanis Andreas:** Writing – review & editing, Writing – original draft. **Mintis Dimitris G.:** Writing – review & editing, Writing – original draft. **Riudavets-Puig Rafael:** Writing – review & editing, Writing – original draft, Methodology. **Varsou Dimitra-Danai:** Writing – review & editing, Writing – original draft. **Papavasileiou Konstantinos D.:** Writing – review & editing, Writing – original draft. **Kolokathis Panagiotis D.:** Writing – review & editing, Writing – original draft. **Virmani Ishita:** Writing – review & editing, Writing – original draft, Methodology. **Di Lieto Emanuele:** Writing – original draft, Writing – review & editing. **Saarimäki Laura Aliisa:** Writing – original draft, Writing – review & editing. **Morikka Jack:** Writing – review & editing, Writing – original draft. **Tsiros Periklis:** Writing – review & editing, Writing – original draft, Methodology. **Greco Dario:** Writing – review & editing, Writing – original draft, Conceptualization, Funding acquisition, Methodology. **Schaffert Alexandra:** Writing – review & editing, Writing – original draft, Funding acquisition, Methodology. **Torres Maia Marcella:** Writing – review & editing, Writing – original draft, Methodology, Visualization. **Afantitis Antreas:** Writing – review & editing, Writing – original draft, Funding acquisition, Methodology. **Petry Romana:** Writing – review & editing, Writing – original draft. **Teodoro Martinez Diego Stéfani:** Writing – review & editing, Funding acquisition, Writing – original draft. **Serra Angela:** Writing – review & editing, Writing – original draft, Conceptualization, Visualization, Methodology, Project administration, Supervision. **Zouraris Dimitrios:** Writing – review & editing, Writing – original draft, Visualization, Methodology. **Peijnenburg Willie:** Writing – review & editing, Funding acquisition, Writing – original draft, Methodology. **Park Seung-Geun:** Writing – review & editing, Writing – original draft. **Sarimveis Haralambos:** Writing – review & editing, Writing – original draft, Funding acquisition, Methodology. **Ha Seung Min:** Writing – review & editing, Writing – original draft. **Paparella Martin:** Writing – review & editing, Writing – original draft, Funding acquisition, Methodology. **Gerelkhuu Zayakhuu:** Writing – review & editing, Writing – original draft. **Lynch Iseult:** Writing – review & editing, Writing – original draft, Funding acquisition, Methodology. **Yoon Tae Hyun:** Writing – review & editing, Funding acquisition, Writing – original draft. **Wick Peter:** Writing – review & editing, Funding acquisition, Writing – original draft, Methodology. **Lindner Gottlieb Georg:** Writing – review & editing, Funding acquisition, Writing – original draft. **Exner Thomas E.:** Writing – review & editing, Funding acquisition, Writing – original draft, Methodology. **Sergent Jacques-Aurélien:** Writing – review & editing, Funding acquisition, Writing – original draft. **Dondero Francesco:** Writing – review & editing, Funding acquisition, Writing – original draft, Methodology. **Gheorghe L. Cristiana:** Writing – review & editing, Writing – original draft. **Serchi Tommaso:** Writing – review & editing, Funding acquisition, Writing – original draft, Methodology. **Bradford Laura-Jayne A.:** Writing – review & editing, Writing – original draft. **Winkler David A.:** Writing – review & editing, Funding acquisition, Writing – original draft. **Isigonis Panagiotis:** Writing – review & editing, Writing – original draft. **Cambier Sébastien:** Writing – review & editing, Writing – original draft. **Marvuglia Antonino:** Writing – review & editing, Methodology, Writing – original draft. **Sarigiannis Dimosthenis A.:** Writing – review & editing, Funding acquisition, Writing – original draft. **Friedrichs Steffi:** Writing – review & editing, Funding acquisition, Writing – original draft. **Seitz Christian:** Writing – review & editing, Writing – original draft. **Gutierrez Tomas Navarrete:** Writing – review & editing, Methodology, Writing – original draft. **Nikiforou Fotini:** Writing – review & editing, Writing – original draft, Methodology. **Karakoltzidis Achilleas:** Writing – review & editing, Methodology, Writing – original draft. **Karakitsios Spyros:** Writing – review & editing, Funding acquisition, Writing – original draft.

## Declaration of Competing Interest

The authors declare that they have no known competing financial interests or personal relationships that could have appeared to influence the work reported in this paper.
